# The Platelet-to-Hemoglobin Ratio as a Prognostic Marker in Patients with Diabetes Mellitus and Acute Coronary Syndrome

**DOI:** 10.3390/jcm14196780

**Published:** 2025-09-25

**Authors:** Christos Kofos, Panagiotis Stachteas, Barbara Fyntanidou, Andreas S. Papazoglou, Athanasios Samaras, Athina Nasoufidou, Aikaterini Apostolopoulou, Paschalis Karakasis, Alexandra Arvanitaki, Marios G. Bantidos, Dimitrios V. Moysidis, Nikolaos Stalikas, Dimitrios Patoulias, Marios Sagris, Apostolos Tzikas, George Kassimis, Nikolaos Fragakis, Efstratios Karagiannidis

**Affiliations:** 1 Second Department of Cardiology, General Hospital ‘Hippokration’, Aristotle University of Thessaloniki, Konstantinoupoleos 49, 54642 Thessaloniki, Greece; chriskofos21@gmail.com (C.K.);; 2 Department of Emergency Medicine, AHEPA University Hospital, 54636 Thessaloniki, Greeceaapostoo@auth.gr (A.A.); mpantidos@auth.gr (M.G.B.); 3 Athens Naval Hospital, 11521 Athens, Greece; 4 Medical School, Aristotle University of Thessaloniki, 54124 Thessaloniki, Greece; 5 Cardiovascular Center, AZORG Ziekenhuis, Moorselbaan 164, 9300 Aalst, Belgium; 6 Second Propaedeutic Department of Internal Medicine, Faculty of Medicine, School of Health Sciences Aristotle, University of Thessaloniki, 54124 Thessaloniki, Greece; 7 1st Department of Cardiology, Hippokration General Hospital, National and Kapodistrian University of Athens, 11527 Athens, Greece

**Keywords:** platelet-to-hemoglobin ratio, PHR, acute coronary syndrome, diabetes mellitus, prognostic marker, mortality, risk stratification

## Abstract

**Background:** The platelet-to-hemoglobin ratio (PHR) has emerged as a potential prognostic marker in various cardiovascular contexts, but its role in acute coronary syndrome (ACS), particularly among patients with diabetes mellitus (DM), remains unclear. **Methods:** In this retrospective cohort study, 843 ACS patients admitted to the 2nd Cardiology Department at Hippokration Hospital of Thessaloniki, Greece, between 2017 and 2023 were evaluated. PHR was calculated from admission complete blood counts. The primary endpoint was all-cause mortality during a median follow-up of 25 months. Multivariate logistic and Cox regression analyses, receiver operating characteristic (ROC) curves, Kaplan–Meier survival analyses, and restricted cubic spline (RCS) models were employed, with subgroup analyses by DM status. **Results:** Higher PHR was independently associated with increased mortality in the overall cohort (adjusted hazard ratio [aHR] 1.35, *p* < 0.001). This association showed stronger predictive value in DM patients, reflected in both a higher aHR (1.52 vs. 1.36 in non-DM patients, *p* < 0.001 and *p* = 0.018, respectively) and superior discriminative performance on ROC analysis (AUC 0.707 vs. 0.600 overall, *p* = 0.0006). Kaplan–Meier analysis confirmed poorer survival in high-PHR groups, especially in DM patients. RCS analysis revealed a J-shaped relationship, with risk increasing markedly beyond PHR values of 2.2. **Conclusions:** PHR is an independent predictor of long-term mortality in ACS, with greater prognostic significance in DM patients. Its simplicity, low cost, and availability from routine blood tests make it a promising tool for risk stratification in ACS.

## 1. Introduction

Acute coronary syndrome (ACS) remains a leading cause of morbidity and mortality worldwide, despite advances in diagnostic and therapeutic strategies [1]. Risk stratification in ACS is essential for optimizing management decisions and improving patient outcomes [2]. An expanding body of evidence highlights that established prognostic tools in ACS management rely on a combination of clinical assessment, biochemical markers, and imaging parameters. Current biomarkers, such as cardiac troponins and natriuretic peptides, play a pivotal role in risk stratification and therapeutic guidance.

Nevertheless, there remains significant interest in discovering novel, cost-effective biomarkers that could enhance prognostic accuracy and optimize individualized care, particularly in high-risk populations [3]. While established cardiac biomarkers remain central to the diagnosis and risk stratification in ACS, growing interest has also turned toward hematological indices derived from routine complete blood counts [4,5]. These easily accessible and cost-effective markers are increasingly recognized for their ability to reflect systemic inflammation, thrombosis, and oxygen-carrying capacity, three closely linked processes that are deeply involved in the pathophysiology of ACS [6].

The platelet-to-hemoglobin ratio (PHR), a biomarker calculated by dividing platelet count by hemoglobin concentration, has recently emerged as a potential prognostic marker in various cardiovascular and non-cardiovascular settings, including rheumatoid arthritis, where it has been investigated as a marker of systemic inflammation and disease activity [7,8]. Platelet activation plays a central role in thrombus formation following atherosclerotic plaque rupture [9]. At the same time, anemia has been independently associated with adverse outcomes in ACS due to impaired oxygen delivery and increased myocardial workload [10]. The PHR may thus integrate two opposing biological forces, thrombotic potential and oxygen-carrying capacity, into a single parameter. However, the prognostic utility of PHR in ACS remains understudied, and its clinical relevance has yet to be validated across different patient subgroups.

Diabetes mellitus (DM) is a well-established risk factor for both the development and progression of coronary artery disease (CAD) and is frequently associated with more diffuse atherosclerosis, endothelial dysfunction, and prothrombotic states [11,12]. Furthermore, diabetic patients often present with atypical symptoms, delayed diagnoses, and poorer outcomes following ACS [13]. Given that both platelet reactivity and anemia are commonly altered in individuals with DM [14,15], the prognostic behavior of PHR may differ between diabetic and non-diabetic populations. Understanding whether diabetic status modifies the prognostic value of PHR in ACS could help refine risk assessment in this heterogeneous patient group.

In this study, we aimed to evaluate the prognostic significance of the platelet-to-hemoglobin ratio in patients with ACS. As a secondary study objective, we also investigated whether the predictive performance of PHR varies according to the presence or absence of DM.

## 2. Methods

### 2.1. Study Population

This retrospective cohort study included consecutive patients admitted to the 2nd Cardiology Department at Hippokration Hospital of Thessaloniki, Greece, over the period 2017 to 2023. Eligible participants were adults aged 18 years or older with a confirmed diagnosis of ACS. ACS diagnosis was established according to the Fourth Universal Definition of Myocardial Infarction (2018), incorporating clinical presentation, ECG changes, and cardiac biomarker elevation [16].

Ethical approval for this study was granted by the Hippokration Hospital Ethics Committee. In line with the retrospective nature of the study, the need for individual informed consent was waived, and all procedures adhered to the ethical standards of the Declaration of Helsinki [17].

### 2.2. Study Endpoint and Follow-Up Procedures

The primary study endpoint was to evaluate the prognostic value of the PHR for predicting all-cause mortality in ACS patients, with a secondary study endpoint to identify any existing differences between individuals with and without DM. All-cause mortality was defined as death from any cause during a median follow-up period of approximately two years. Follow-up information was obtained through review of electronic medical records, supplemented by telephone contact with patients or their relatives when necessary. Survival status was attained by cross-referencing hospital records with national mortality registries to ensure accuracy and completeness of the outcome data.

### 2.3. Statistical Analysis

Baseline characteristics were summarized for the overall study population. Continuous variables were reported as means ± standard deviations if normally distributed, or as medians with interquartile ranges (IQR) when distributions were skewed. Categorical variables were expressed as frequencies and percentages. Patients were classified according to the presentation of ACS as STEMI, NSTEMI, or unstable angina. For descriptive purposes, we summarized the distribution of ACS subtypes, the prevalence of DM within each subgroup, and PHR values overall and by DM status.

Logistic regression analyses were performed initially on the entire cohort to explore associations between PHR and mortality, adjusting for potential confounders such as demographic factors (age, sex), clinical comorbidities (hypertension, dyslipidemia, chronic kidney disease, heart failure history, atrial fibrillation), laboratory values at admission (serum creatinine, high-sensitivity troponin I) prescribed medications at admission, such as anticoagulant use (including direct oral anticoagulants (DOACs), low-molecular-weight heparin (LMWH), and unfractionated heparin (UFH)), statin use and β-blockers, and smoking status. Subgroup analyses were then conducted to compare outcomes in diabetic versus non-diabetic subgroups. Unadjusted and adjusted odds ratios [(a)ORs] along with 95% confidence intervals (CIs) are reported for each analysis.

Receiver operating characteristic (ROC) curve analysis assessed the discriminative performance of PHR in mortality prediction. Area under the curve (AUC) values were calculated for the overall cohort as well as stratified by diabetic status. Comparisons of AUCs between subgroups were performed using the DeLong test to identify any significant differences in predictive accuracy.

Time-to-event outcomes were evaluated using Cox proportional hazards regression and Kaplan–Meier (KM) survival analysis. Follow-up time was truncated at the 90th percentile to minimize curve instability from sparse late events. Univariate and multivariate Cox models assessed the association between PHR and all-cause mortality in the total cohort and in subgroups stratified by diabetes status. Adjusted models included age, sex, history of heart failure, hypertension, diabetes mellitus, dyslipidemia, smoking status, chronic kidney disease, atrial fibrillation, serum creatinine, high-sensitivity troponin I, use of anticoagulants, and use of statins. Results are reported as hazard ratios (HRs) and adjusted HRs (aHRs) with 95% confidence intervals (CIs).

For the KM analyses, patients were first dichotomized into high and low PHR groups using the optimal cut-off determined by Youden’s Index. Additional KM analyses were performed by categorizing PHR into quartiles, followed by pairwise log-rank tests with Bonferroni correction, and a focused comparison of the highest quartile (Q4) versus the combined lower three quartiles (Q1–Q3). All KM analyses were conducted separately for the total, diabetic, and non-diabetic populations. Survival differences were evaluated using the log-rank test.

To explore potential non-linear relationships between PHR and mortality risk, restricted cubic spline (RCS) regression analyses [18] were applied within Cox models, employing four knots selected based on model fit criteria. This approach allowed detailed modeling of mortality risk patterns across the spectrum of PHR values.

All statistical analyses were conducted using IBM SPSS Statistics (version 28.0) and R software (version 4.4.2). A *p*-value < 0.05 was considered statistically significant.

## 3. Results

A total of 843 patients were included in the study, of whom 27.2% were women, with a mean age of 64.4 ± 13.0 years. During a median follow-up of 25 months (IQR: 24–26 months), 158 (17.7%) patients died. The prevalence of DM was 26.7%. Compared with non-DM patients, those with DM were significantly older, had higher PHR values and serum creatinine levels, and a greater prevalence of hypertension, dyslipidemia, chronic kidney disease, and atrial fibrillation. Diabetic patients were also more often treated with anticoagulants, statins, and beta-blockers, while smoking and family history of cardiovascular disease were more common among non-DM (Table 1).

When stratified by ACS subtype, 38.1% of patients presented with STEMI, 29.7% with NSTEMI, and 32.2% with unstable angina. The prevalence of DM varied across subtypes, ranging from 21.0% in STEMI to 27.8% in NSTEMI and 32.5% in unstable angina. Mean PHR values were 1.84 ± 0.76 in STEMI, 2.02 ± 0.83 in NSTEMI, and 1.86 ± 0.72 in unstable angina. Within DM patients, PHR values were consistently higher (STEMI: 1.92 ± 0.76, NSTEMI: 2.38 ± 1.21, unstable angina: 1.96 ± 0.85) compared with their non-DM counterparts (STEMI: 1.82 ± 0.75, NSTEMI: 1.88 ± 0.58, unstable angina: 1.82 ± 0.66).

In the univariate binary logistic regression model analyzing the entire study population, higher PHR (as a continuous variable) was significantly associated with increased risk of all-cause mortality (OR: 1.61, 95% CI: 1.30–1.98, *p* < 0.001). In the multivariate model, after adjusting for relevant confounders including age, gender, hypertension, dyslipidemia, smoking status, chronic kidney disease, atrial fibrillation, heart failure, use of anticoagulants, use of beta-blockers, serum creatinine, admission troponin, and DM, PHR remained an independent predictor of mortality (aOR: 1.67, 95% CI: 1.29–2.17, *p* < 0.001) (Table 2). Among patients with DM, PHR was significantly associated with mortality in both univariate (OR: 2.08, 95% CI: 1.48–3.03, *p* < 0.001) and multivariate analyses (aOR: 1.96, 95% CI: 1.34–3.02, *p* = 0.001) (Supplementary Table S1).

In the non-DM subgroup, PHR was not significantly associated with mortality in univariate analysis (OR: 1.24, 95% CI: 0.92–1.65, *p* = 0.145). However, after adjustment for relevant clinical variables, PHR became an independent predictor of mortality (aOR: 1.41, 95% CI: 1.01–2.00, *p* = 0.047) (Supplementary Table S2).

### 3.1. ROC Curve Analysis

**Figure 1:**
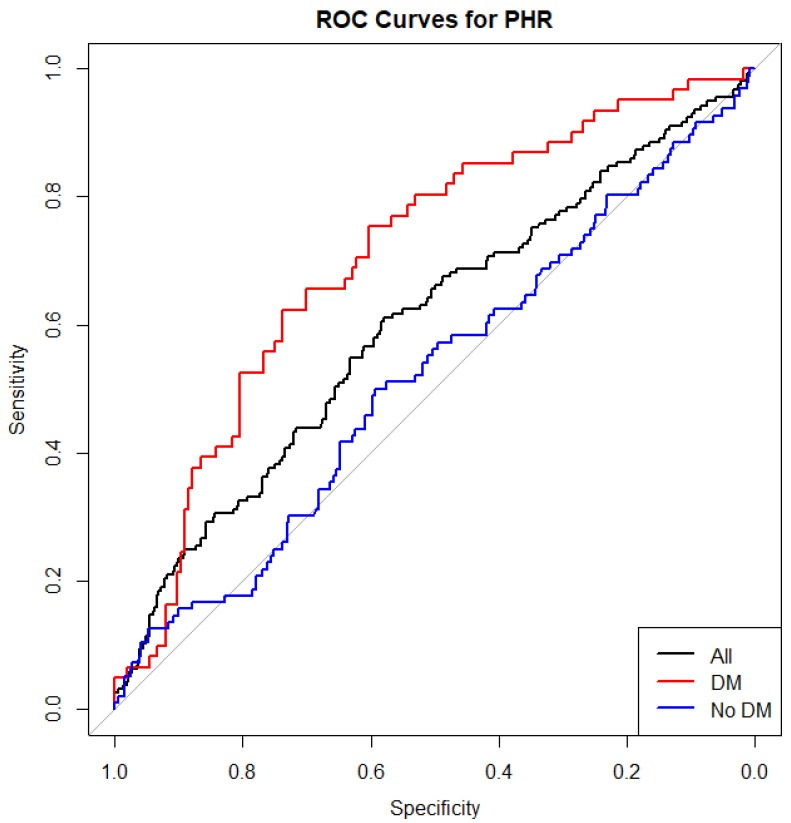
Receiver Operating Characteristic (ROC) curve illustrating the discriminative ability of PHR for predicting all-cause mortality in the total study population (black line), DM patients (red line), and non-DM patients (blue line).

In the total population, the AUC was 0.600 (95% C.I.: 0.549–0.651), indicating modest discriminatory capacity. Using Youden’s Index, the optimal cutoff point for PHR was identified at 1.815. Subgroup analyses were then conducted based on the presence or absence of DM. Among patients with DM, the AUC was 0.707 (95% CI: 0.631–0.782), whereas in non-diabetic patients, the AUC was 0.519 (95% CI: 0.454–0.584), demonstrating a very weak and statistically insignificant ability to distinguish between survivors and non-survivors. This result suggests that the model’s predictions are not reliable for classification purposes in this subgroup (Figure 1). The difference in AUCs between the two subgroups was statistically significant (DeLong’s test: *p* = 0.0006), indicating a notable interaction between PHR and diabetic status in terms of prognostic value.

### 3.2. Survival Analysis

**Figure 2:**
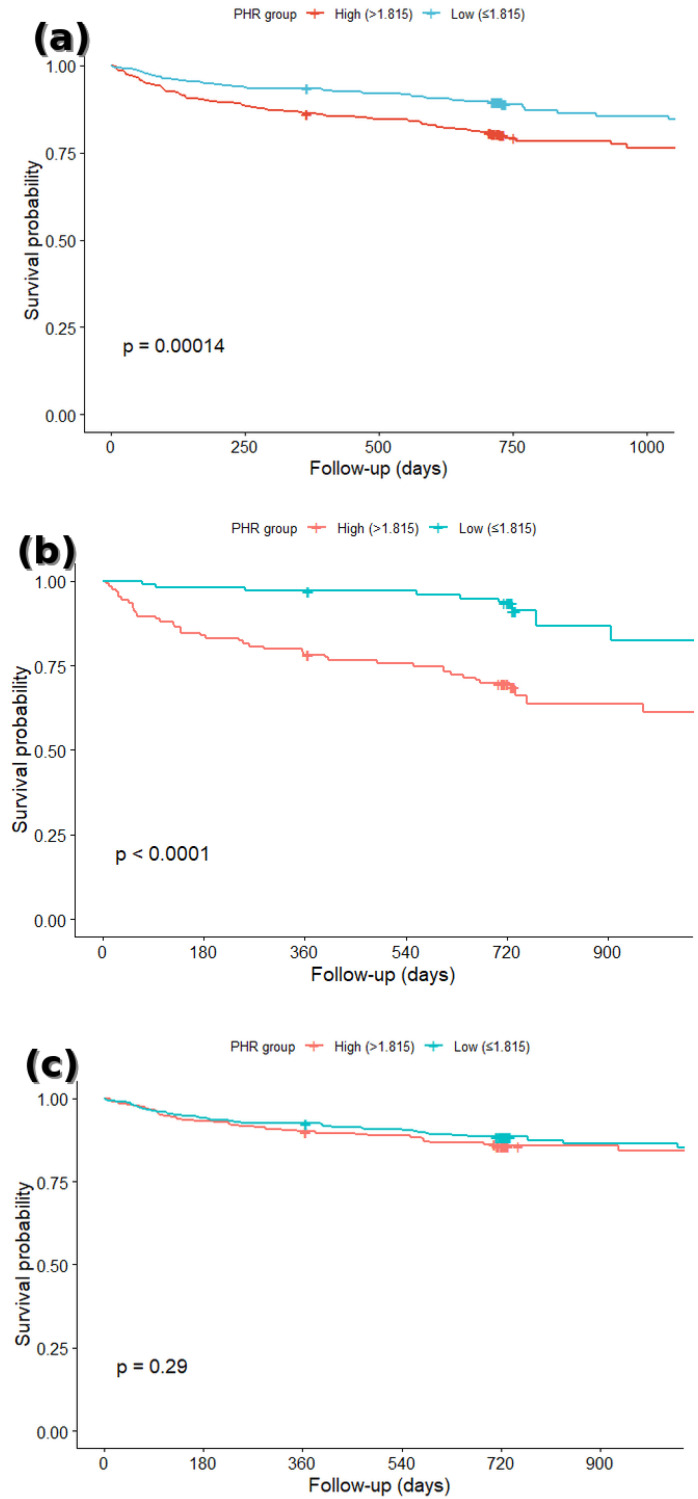
Kaplan–Meier survival curves comparing patients with high and low PHR values, using the optimal cut-off value derived from Jouden’s index. (**a**) Entire cohort: high PHR (red line) vs. low PHR (blue line). (**b**) Patients with diabetes mellitus: high PHR (blue line) vs. low PHR (red line). (**c**) Patients without diabetes mellitus: high PHR (blue line) vs. low PHR (red line).

Using the optimal PHR cut-off value of 1.815 (derived from Youden’s Index), patients were stratified into low and high PHR groups. After truncating follow-up at the 90th percentile to avoid instability in the tail of the curves, Kaplan–Meier analysis in the total population showed that the high-PHR group had significantly worse survival than the low-PHR group (Figure 2a) (log-rank *p* = 0.00014).

In subgroup analyses, this association was pronounced in patients with DM, where the high-PHR group exhibited notably lower survival compared with the low-PHR group (Figure 2b) (log-rank *p* < 0.0001). In contrast, no significant survival difference between high- and low-PHR groups was observed among non-diabetic patients (Figure 2c) (log-rank *p* = 0.29). These findings reinforce a potential modifying effect of diabetic status on the prognostic value of PHR in ACS.

To further explore the prognostic relationship, patients were also classified into quartiles according to PHR distribution. In the total population, survival differed significantly across quartiles (overall log-rank *p* = 0.02), with the highest quartile (Q4) showing the poorest outcomes. In unadjusted Kaplan–Meier pairwise comparisons, Q4 survival was significantly worse than Q2 (*p* = 0.045) and showed a strong trend toward worse survival compared with Q1 (*p* = 0.074). However, after adjusting for potential confounders in Cox regression, Q4 was associated with an 84% higher risk of death compared with Q1 (aHR 1.84, 95% CI 1.14–2.98, *p* = 0.013), indicating that the association became more pronounced when accounting for other risk factors.

Quartile analysis in DM patients showed an even more pronounced gradient in survival (overall log-rank *p* = 0.0001). Both Q3 and Q4 were associated with significantly higher mortality compared with Q1, with Q4 patients experiencing more than a threefold increase in risk (aHR 3.59, 95% CI 1.53–8.42, *p* = 0.003). Conversely, among non-diabetic patients, survival curves across quartiles were nearly overlapping (overall log-rank *p* = 0.7), and no quartile was significantly associated with mortality in the Cox regression model.

**Figure 3:**
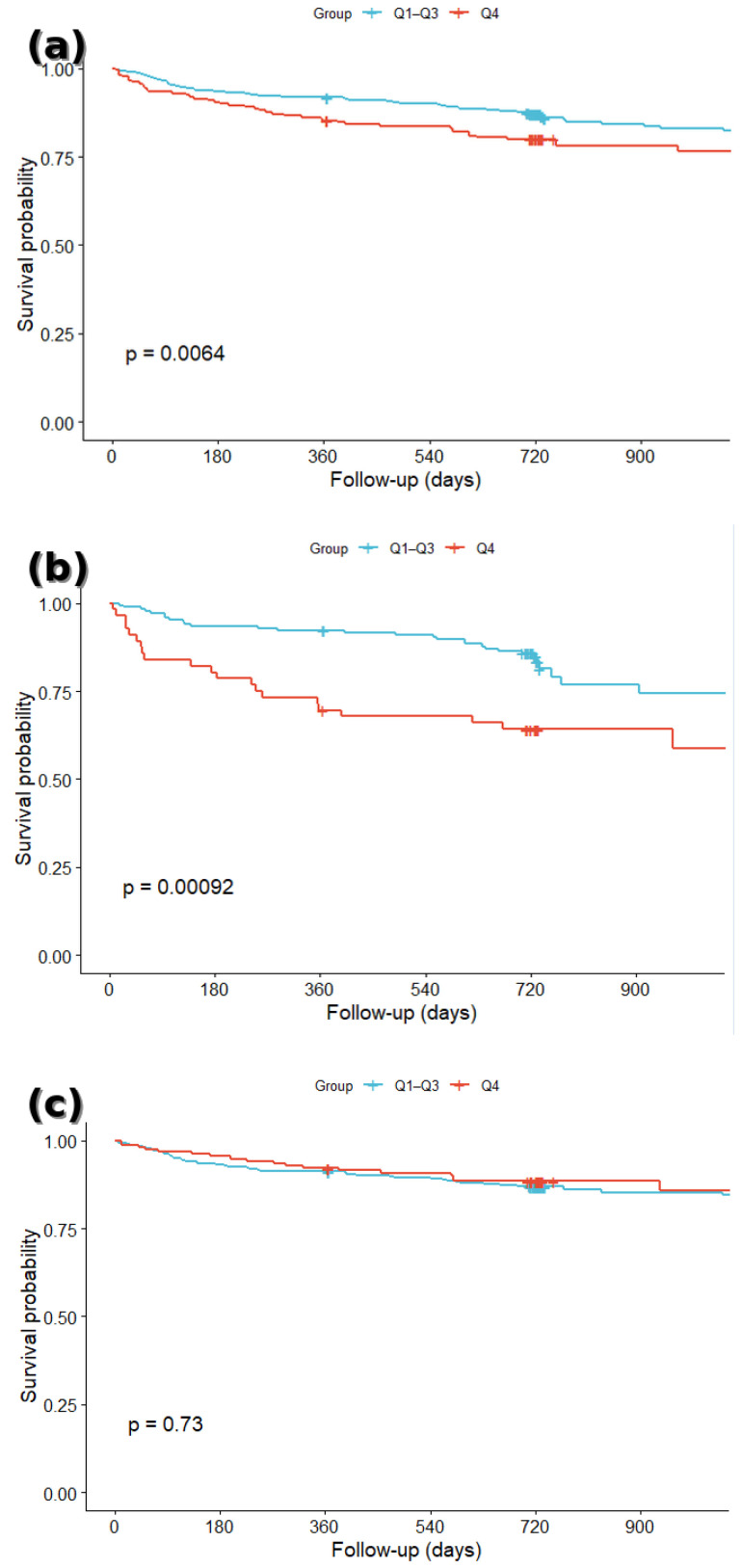
Kaplan–Meier survival curves comparing patients in the highest PHR quartile (Q4) with those in the lower three quartiles combined (Q1–Q3). (**a**) Total population, (**b**) patients with diabetes mellitus, and (**c**) patients without diabetes mellitus. Survival differences were assessed using the log-rank test.

For clinical interpretability, we further compared Q4 with the combined lower three quartiles (Q1–Q3). In the total population, Q4 patients had significantly worse survival than Q1–Q3 (Figure 3a) (log-rank *p* = 0.006), corresponding to a 63% higher mortality risk (aHR 1.63, 95% CI 1.14–2.33). In diabetic patients, the difference was more pronounced (Figure 3b) (log-rank *p* = 0.0009), with Q4 showing a 2.45-fold higher mortality risk (aHR 2.45, 95% CI 1.42–4.23). In non-diabetic patients, survival was similar between Q4 and Q1–Q3 (Figure 3c) (log-rank *p* = 0.73), with no significant difference in mortality risk (aHR 0.91, 95% CI 0.55–1.53).

In the overall cohort, univariate Cox regression analysis demonstrated that higher PHR (as a continuous variable) was significantly associated with increased risk of all-cause mortality (HR 1.414, 95% CI 1.224–1.633, *p* < 0.001). This association remained robust after adjustment for potential confounders, including age, gender, history of heart failure, hypertension, dyslipidemia, DM, smoking status, chronic kidney disease, atrial fibrillation, use of anticoagulants, use of statins, high-sensitive Troponin I, and serum creatinine. In the adjusted model, PHR remained an independent predictor of mortality (aHR 1.406, 95% CI 1.210–1.634, *p* < 0.001) (Table 3).

Among patients with diabetes mellitus, PHR was a strong and consistent predictor of mortality in both univariate and multivariate analyses. Univariate analysis showed that each unit increase in PHR was associated with a 56% higher mortality risk (HR = 1.559, 95% CI: 1.314–1.850, *p* < 0.001), and this effect persisted after adjustment (aHR = 1.521, 95% CI: 1.237–1.869, *p* < 0.001) (Supplementary Table S3).

In contrast, among patients without diabetes, univariate Cox regression did not reveal a significant association between PHR and mortality (HR 1.175, 95% CI 0.909–1.520, *p* = 0.218). However, in the multivariate analysis, higher PHR was independently associated with increased mortality risk (aHR 1.361, 95% CI 1.053–1.758, *p* = 0.018) (Supplementary Table S4).

### 3.3. Restricted Cubic Spline (RCS) Analysis

**Figure 4:**
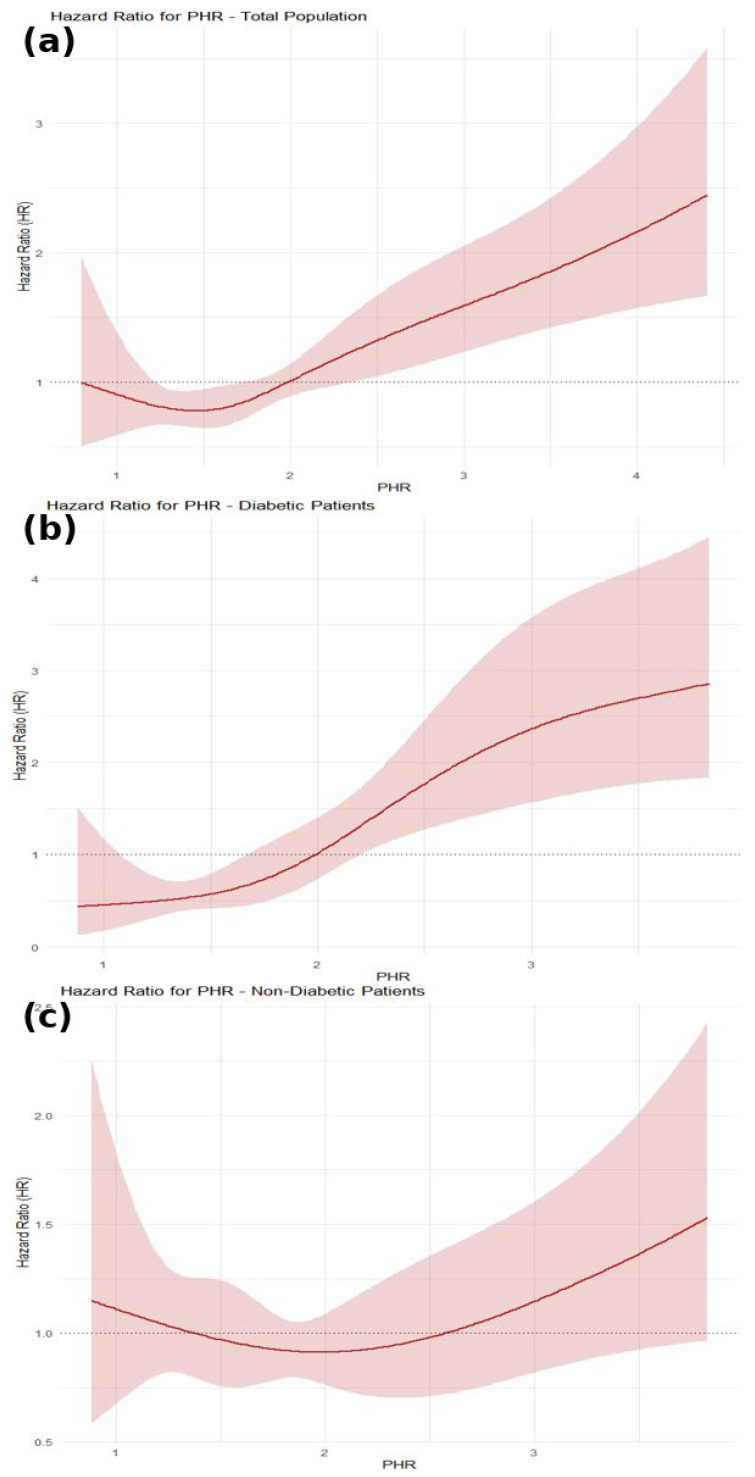
Restricted cubic spline plots showing the association between PHR and the hazard of all-cause mortality in patients with ACS. (**a**) Overall population, (**b**) patients with diabetes mellitus, and (**c**) patients without diabetes mellitus.

The RCS analysis revealed a non-linear, J-shaped association between the PHR and all-cause mortality in the overall population. In the total population, PHR values between 1.3 and 1.8 were associated with a statistically significant lower mortality risk, while values above 2.2 were associated with a significantly higher mortality risk (Figure 4a). In DM patients, the RCS curve showed no statistically significant reduction in mortality risk across lower PHR values. Mortality risk began to rise progressively from approximately PHR = 2.0 and became statistically significant beyond PHR = 2.2, indicating that higher PHR values are associated with increased risk in this subgroup (Figure 4b). In non-diabetic patients, the spline curve remained relatively flat at lower PHR values, with no meaningful change in HR until approximately PHR = 1.9, after which the hazard ratio increased sharply and reached statistical significance around PHR = 2.6 (Figure 4c). These findings underscore the prognostic significance of PHR, particularly in individuals with DM.

## 4. Discussion

In this retrospective cohort study, we demonstrated that an elevated PHR was significantly associated with an increased risk of long-term all-cause mortality in patients presenting with ACS. Importantly, the prognostic performance of PHR appeared more robust among DM individuals, suggesting that this simple hematological index may be particularly useful for risk stratification in this high-risk subgroup. This association remained relevant after adjusting for key clinical variables. The discriminative ability of PHR was supported by ROC analysis, and a non-linear relationship between PHR and adverse outcomes was identified using RCS modeling, suggesting a threshold effect beyond which risk escalates steeply. The discriminative ability of PHR was modest in the overall cohort, but its predictive performance was enhanced among diabetic patients, indicating that PHR may have particular clinical value in high-risk groups.

Our findings are consistent with and expand upon previous studies examining PHR in cardiovascular populations. Işık and Soner reported that elevated PHR independently predicted in-hospital mortality in ST-elevation myocardial infarction (STEMI) patients, with an AUC of 0.72, which is comparable to our own ROC performance [19]. Kunming Bao’s work in CAD patients with heart failure showed that higher PHR levels were linked with long-term all-cause mortality [7], while Ying-Ying Zheng and colleagues found a similar association in post-percutaneous coronary intervention (PCI) patients [20]. Notably, Bao also employed RCS analysis, revealing a J-shaped curve, which aligns with the non-linear trend we observed [7].

It is also important to distinguish between chronic coronary syndrome (CCS) and de novo suspected myocardial ischemia, as these populations differ in both clinical presentation and prognostic implications. CCS patients usually represent a more stable phenotype with established atherosclerosis and recurrent ischemic burden [21], while de novo ischemia describes first-time presentations where ischemia is suspected but coronary disease has not been previously documented [22]. These distinctions highlight the heterogeneity across coronary disease presentations and provide context for interpreting the prognostic role of hematological indices such as the PHR.

Several biological mechanisms may explain the prognostic role of PHR in ACS. Thrombocytosis is a known marker of inflammation and platelet-driven hypercoagulability, both of which contribute to plaque instability and thrombosis [23]. Meanwhile, anemia may reflect chronic disease burden, impaired oxygen delivery, and neurohormonal activation, all of which contribute to adverse cardiovascular outcomes [24]. Beyond these individual effects, platelet activity and hemoglobin levels may influence each other in the acute setting. Reduced hemoglobin can enhance platelet reactivity through hypoxia-driven pathways [25], while inflammatory mediators that elevate platelet count can simultaneously suppress erythropoiesis [26]. The PHR thus integrates two distinct yet combined pathophysiological processes, offering a more detailed overview of cardiovascular risk.

In addition, lifelong exercise practice has been shown to exert profound anti-inflammatory and metabolic benefits, mitigating plaque progression and improving cardiovascular outcomes [27]. Considering that PHR reflects both thrombotic potential and oxygen-carrying capacity, incorporating lifestyle factors such as regular exercise into risk assessment models could further enhance their predictive power. Future research should therefore explore the interaction between physical activity, PHR, and long-term prognosis in ACS, as this may provide a more comprehensive view of patient risk.

DM appears to modify the prognostic significance of PHR, as reflected by the stronger association observed in diabetic patients within our cohort. The chronic inflammatory environment and enhanced platelet reactivity, characteristic of DM, promote thrombocytosis and contribute to vascular complications [28]. Simultaneously, DM-related anemia, often due to chronic kidney disease and impaired erythropoiesis [29], may further elevate PHR values. Therefore, in patients with DM, PHR likely reflects a combined risk linked to both blood-related abnormalities and metabolic dysfunction, highlighting its potential as an important prognostic marker in this population.

Beyond its biological plausibility, the clinical appeal of PHR lies in its simplicity, availability, and cost-effectiveness. Unlike other biomarkers that require specialized assays [30], PHR can be calculated from a standard complete blood count, which is routinely performed in all ACS patients upon admission. This could facilitate its integration into existing risk models, potentially improving early risk stratification without incurring additional costs.

Complementary to PHR, several other hematological indices have been studied as prognostic tools in cardiovascular disease. For instance, the neutrophil-to-lymphocyte ratio and platelet-to-lymphocyte ratio have both been associated with adverse outcomes in ACS populations, reflecting the contribution of systemic inflammation and platelet activity to cardiovascular risk [31,32]. Similarly, red cell distribution width has been linked to increased mortality and poor outcomes in patients with coronary artery disease [33]. Together, these findings support the concept that simple hematological markers derived from routine laboratory testing can provide valuable prognostic information. Within this framework, our study extends prior work by demonstrating that PHR, an easily available index integrating platelet activity and hemoglobin status, is independently associated with long-term mortality, with particularly strong predictive performance in DM patients.

### Limitations

Despite the strengths, our study has several limitations. First, its retrospective design is inherently subject to selection bias and unmeasured confounders. In particular, information on revascularization strategies or detailed antiplatelet therapy was not available, both of which are important determinants of outcomes in ACS and may have confounded our results. Second, PHR was measured only upon admission, and we did not assess serial changes or long-term trends. Third, although our model included important covariates, we lacked data on inflammatory biomarkers (e.g., CRP or IL-6) and iron status that could further clarify the mechanisms linking PHR with outcomes. Furthermore, conducting the study within a single institution limits the generalizability of our findings to other populations or clinical settings. In addition, our endpoint was all-cause mortality, which, although robust and unbiased, may reduce specificity compared with cardiovascular-specific outcomes such as major adverse cardiovascular events or cardiovascular death. Moreover, external validation in an independent prospective cohort is necessary before PHR can be routinely adopted as a clinical prognostic tool.

## 5. Conclusions

In conclusion, in this retrospective cohort of patients with ACS, higher PHR was independently associated with increased long-term mortality, with the prognostic signal being superior among individuals with DM. The observed non-linear relationship and differential predictive performance by diabetic status suggest that PHR may capture distinct pathophysiological processes in this subgroup. As an inexpensive and readily obtainable parameter from routine blood tests, PHR holds promise as a useful tool for risk stratification in ACS, especially in diabetic patients. Prospective studies and external validation in diverse populations are warranted to confirm these findings and clarify the role of PHR in clinical decision-making.

## Figures and Tables

**Table 1:** Baseline Characteristics of the Study Population by Diabetes Mellitus Status. *p-values refer to comparisons between non-DM and DM groups*.

Characteristic	Total (*n* = 843)	Non-DM (*n* = 618)	DM (*n* = 225)	*p*-Value ^1^
**Age, years**	64.4 ± 13.0	62.7 ± 13.1	69.0 ± 11.6	<0.001
**PHR**	1.90 ± 0.78	1.84 ± 0.69	2.08 ± 0.95	<0.001
**Serum creatinine, mg/dL**	1.19 ± 1.00	1.12 ± 0.82	1.40 ± 1.37	<0.001
**High-sensitivity troponin I**	1440.8 ± 3194.4	1546.9 ± 3404.0	1155.0 ± 2531.3	0.123
**Male sex, *n* (%)**	614 (72.8)	460 (74.4)	154 (68.4)	0.101
**Heart failure, *n* (%)**	24 (2.8)	11 (2.0)	13 (6.2)	0.005
**Hypertension, *n* (%)**	413 (49.0)	245 (39.7)	168 (74.7)	<0.001
**Dyslipidemia, *n* (%)**	217 (25.8)	128 (20.8)	89 (39.6)	<0.001
**Chronic kidney disease, *n* (%)**	55 (7.2)	23 (4.1)	32 (15.3)	<0.001
**Atrial fibrillation, *n* (%)**	72 (9.4)	42 (7.5)	30 (14.4)	0.006
**Family history of CVD, *n* (%)**	124 (14.8)	102 (16.6)	22 (9.9)	0.021
**Smoking, *n* (%)**	379 (45.1)	297 (48.2)	82 (36.6)	0.004
**Anticoagulant use, *n* (%)**	160 (19.1)	102 (16.6)	58 (25.8)	0.004
**Statin use, *n* (%)**	404 (48.4)	272 (44.6)	132 (58.9)	<0.001
**Beta-blocker use, *n* (%)**	394 (47.3)	271 (44.5)	123 (54.9)	0.010

^1^ Welch two-sample *t*-test for continuous variables; Pearson’s chi-square test for categorical variables.

**Table 2:** Univariate and multivariate logistic regression analysis for PHR in association with mortality in the total population.

Variable	Univariate OR (95% CI)	*p*-Value (Univariate)	Multivariate aOR (95% CI)	*p*-Value (Multivariate)
PHR	1.607 (1.304–1.981)	<0.001	1.672 (1.289–2.169)	<0.001
Heart Failure	4.038 (1.806–9.029)	<0.001	3.077 (1.286–9.207)	0.014
Gender	0.793 (0.552–1.140)	0.210	1.367 (0.852–2.195)	0.195
Hypertension	0.945 (0.676–1.320)	0.739	0.692 (0.444–1.078)	0.103
Diabetes Mellitus	2.119 (1.492–3.011)	<0.001	1.441 (0.904–2.298)	0.125
Dyslipidemia	0.613 (0.402–0.936)	0.024	0.567 (0.341–0.944)	0.029
Smoking	0.477 (0.333–0.685)	<0.001	0.731 (0.454–1.175)	0.195
Age	1.048 (1.033–1.063)	<0.001	1.029 (1.010–1.049)	0.003
Chronic Kidney Disease	3.868 (2.249–6.651)	<0.001	0.987 (0.432–2.256)	0.976
Atrial Fibrillation	2.083 (1.251–3.468)	0.005	0.889 (0.451–1.750)	0.733
Serum creatinine	1.506 (1.274–1.779)	<0.001	1.277 (1.044–1.563)	0.017
Anticoagulant Use	2.149 (1.473–3.135)	<0.001	1.822 (1.109–2.996)	0.018
Beta-blocker Use	1.492 (1.064–2.092)	0.020	1.078 (0.705–1.648)	0.729
High-sensitive Troponin I	1.002 (1.001–1.003)	0.047	1.002 (1.001–1.003)	0.354

**Table 3:** Univariate and multivariate Cox regression analysis for PHR in association with mortality in the total population.

Variable	Univariate HR (95% CI)	*p*-Value	Multivariate HR (95% CI)	*p*-Value
PHR	1.414 (1.224–1.642)	<0.001	1.406 (1.210–1.634)	<0.001
Heart Failure	3.82 (2.114–6.921)	<0.001	2.864 (1.469–5.582)	0.002
Gender	0.93 (0.672–1.283)	0.649	1.307 (0.875–1.951)	0.191
Hypertension	0.97 (0.721–1.314)	0.838	0.833 (0.573–1.213)	0.341
Diabetes Mellitus	1.629 (1.192–2.227)	0.002	1.298 (0.884–1.907)	0.183
Dyslipidemia	0.856 (0.573–1.269)	0.408	0.930 (0.588–1.472)	0.758
Smoking	0.734 (0.526–1.033)	0.077	0.881 (0.574–1.352)	0.562
Age	1.035 (1.025–1.042)	<0.001	1.019 (1.003–1.036)	0.020
Chronic Kidney Disease	3.086 (2.043–4.701)	<0.001	1.284 (0.686–2.403)	0.435
Atrial Fibrillation	1.724 (1.123–2.646)	0.014	0.949 (0.549–1.639)	0.851
Serum creatinine	1.203 (1.087–1.312)	<0.001	1.164 (1.022–1.324)	0.022
Anticoagulant Use	1.575 (1.127–2.182)	0.008	1.307 (0.663–1.982)	0.207
Statin Use	0.725 (0.531–0.987)	0.035	0.636 (0.444–0.912)	0.014
High-sensitive Troponin I	1.002 (1.001–1.003)	0.004	1.002 (1.001–1.003)	0.098

## Data Availability

Study data will be available upon reasonable request from the corresponding study author (EK).

## Supplementary Materials

The following supporting information can be downloaded at: https://www.mdpi.com/article/10.3390/jcm14196780/s1, Table S1: Univariate and multivariate binary logistic regression analyses for predictors of all-cause mortality during follow-up in patients with diabetes mellitus; Table S2: Univariate and multivariate binary logistic regression analyses for predictors of all-cause mortality during follow-up in patients without diabetes mellitus; Table S3: Univariate and multivariate Cox proportional hazard regression analyses for predictors of all-cause mortality during follow-up in patients with diabetes mellitus; Table S4: Univariate and multivariate Cox proportional hazard regression analyses for predictors of all-cause mortality during follow-up in patients without diabetes mellitus.
