# Supplementary material for: The Platelet-to-Hemoglobin Ratio as a Prognostic Marker in Patients with Diabetes Mellitus and Acute Coronary Syndrome
Source: J Clin Med. 2025 Sep 25;14(19):6780. doi: 10.3390/jcm14196780 (PMC12524964; doi:10.3390/jcm14196780)
Supplement: Supplementary file 1 [file jcm-14-06780-s001.zip › jcm-3846292-supplementary.pdf]

PHR SUPPLEMENTARY TABLES

Variable	Univariate OR (95% CI)	p-value (Univariate)	Multivariate aOR (95% CI)	p-value (Multivariate)
PHR	2.081 (1.475–3.033)	<0.001	1.958 (1.338–3.015)	0.001
Age	1.060 (1.031–1.092)	<0.001	1.034 (0.997–1.074)	0.081
Serum creatinine	1.428 (1.149–1.874)	0.003	1.251 (0.935–1.676)	0.122
High-sensitivity troponin I	1.000 (1.000–1.000)	0.059	1.000 (1.000–1.000)	0.408
Male sex	0.675 (0.376–1.223)	0.191	1.033 (0.474–2.312)	0.936
Heart failure	4.982 (1.649–16.809)	0.006	5.539 (1.265–31.130)	0.032
Hypertension	0.692 (0.376–1.290)	0.239	0.976 (0.423–2.350)	0.956
Dyslipidemia	0.544 (0.290–0.988)	0.050	0.605 (0.274–1.298)	0.204
Chronic kidney disease	2.842 (1.372–5.890)	0.005	0.857 (0.263–2.672)	0.793
Atrial fibrillation	2.939 (1.362–6.359)	0.006	1.815 (0.635–5.164)	0.262
Smoking status	0.389 (0.194–0.737)	0.005	0.593 (0.235–1.424)	0.252
Anticoagulant use	2.356 (1.274–4.345)	0.006	1.443 (0.590–3.459)	0.413
Beta-blocker use	1.496 (0.845–2.688)	0.171	1.204 (0.537–2.731)	0.653

Supplementary Table S1. Univariate and multivariate binary logistic regression analyses for predictors of all-cause mortality during follow-up in patients with diabetes mellitus. Odds ratios (OR) and adjusted odds ratios (aOR) are reported with 95% confidence intervals (CI).

Variable	Univariate OR (95% CI)	p-value (Univariate)	Multivariate aOR (95% CI)	p-value (Multivariate)
PHR	1.240 (0.918–1.647)	0.145	1.414 (1.006–1.999)	0.047
Age	1.038 (1.021–1.056)	<0.001	1.033 (1.011–1.057)	0.004
Serum creatinine	1.488 (1.196–1.906)	<0.001	1.339 (1.007–1.870)	0.057
High-sensitivity Troponin I	1.000 (1.000–1.000)	0.144	1.000 (1.000–1.000)	0.638
Female sex	0.937 (0.589–1.527)	0.789	1.546 (0.839–2.947)	0.172
Heart failure	2.011 (0.434–7.100)	0.309	1.880 (0.359–7.900)	0.410
Hypertension	0.766 (0.489–1.182)	0.234	0.629 (0.359–1.081)	0.098
Dyslipidemia	0.475 (0.240–0.864)	0.022	0.509 (0.233–1.021)	0.071
Chronic kidney disease	3.653 (1.480–8.600)	0.003	1.054 (0.281–3.497)	0.934
Atrial fibrillation	1.279 (0.563–2.632)	0.527	0.475 (0.166–1.218)	0.139
Smoking status	0.574 (0.367–0.887)	0.014	0.734 (0.415–1.293)	0.285
Anticoagulant use	1.844 (1.110–2.998)	0.015	2.033 (1.083–3.742)	0.024
Beta-blocker use	1.364 (0.891–2.093)	0.153	1.104 (0.663–1.837)	0.703

Supplementary Table S2. Univariate and multivariate binary logistic regression analyses for predictors of all-cause mortality during follow-up in patients without diabetes mellitus. Odds ratios (OR) and adjusted odds ratios (aOR) are reported with 95% confidence intervals (CI).

Variable	Univariate HR (95% CI)	p-value (Univariate)	Multivariate aHR (95% CI)	p-value (Multivariate)
PHR	1.559 (1.314–1.850)	<0.001	1.521 (1.237–1.869)	<0.001
Heart Failure	3.513 (1.718–7.183)	<0.001	3.112 (1.342–7.215)	0.008
Gender	0.775 (0.472–1.270)	0.312	1.082 (0.581–2.017)	0.803
Hypertension	0.683 (0.408–1.143)	0.147	0.890 (0.466–1.700)	0.724
Dyslipidaemia	0.843 (0.485–1.466)	0.546	1.141 (0.597–2.183)	0.690
Smoking	0.676 (0.368–1.240)	0.206	0.775 (0.357–1.682)	0.518
Age	1.040 (1.015–1.065)	0.001	1.025 (0.994–1.057)	0.116
Chronic Kidney Disease	2.494 (1.437–4.329)	0.001	1.240 (0.541–2.842)	0.611
Atrial Fibrillation	2.029 (1.143–3.601)	0.016	1.377 (0.656–2.889)	0.398
Serum Creatinine	1.151 (1.022–1.296)	0.020	1.169 (0.954–1.432)	0.133
Anticoagulant Use	1.946 (1.186–3.191)	0.008	1.070 (0.530–2.160)	0.851
Statin Use	0.848 (0.520–1.382)	0.508	0.784 (0.433–1.421)	0.423
High-sensitivity Troponin I	1.000 (1.000–1.000)	0.001	1.000 (1.000–1.000)	0.151

Supplementary Table S3. Univariate and multivariate Cox proportional hazard regression analyses for predictors of all-cause mortality during follow-up in patients with diabetes mellitus. Odds ratios (OR) and adjusted odds ratios (aOR) are reported with 95% confidence intervals (CI).

Variable	Univariate HR (95% CI)	p-value (Univariate)	Multivariate aHR (95% CI)	p-value (Multivariate)
PHR	1.175 (0.909–1.520)	0.218	1.361 (1.053–1.758)	0.018
Heart Failure	2.341 (0.823–6.653)	0.112	1.882 (0.641–5.525)	0.253
Gender	0.945 (0.629–1.421)	0.790	1.457 (0.879–2.415)	0.145
Hypertension	0.855 (0.611–1.195)	0.360	0.782 (0.543–1.127)	0.189
Dyslipidaemia	0.626 (0.391–1.003)	0.051	0.642 (0.392–1.050)	0.077
Smoking	0.640 (0.458–0.895)	0.009	0.796 (0.547–1.158)	0.233
Age	1.046 (1.030–1.062)	<0.001	1.038 (1.021–1.056)	<0.001
Chronic Kidney Disease	3.146 (1.671–5.925)	<0.001	1.784 (0.882–3.607)	0.107
Atrial Fibrillation	1.455 (0.872–2.428)	0.152	1.078 (0.609–1.911)	0.798
Serum Creatinine	1.373 (1.163–1.622)	<0.001	1.220 (1.007–1.477)	0.042
Anticoagulant Use	1.737 (1.181–2.552)	0.005	1.804 (1.178–2.762)	0.006
Statin Use	1.173 (0.823–1.672)	0.377	1.120 (0.774–1.619)	0.547
High-sensitivity Troponin I	1.000 (1.000–1.000)	0.008	1.000 (1.000–1.000)	0.247

Supplementary Table S4. Univariate and multivariate Cox proportional hazard regression analyses for predictors of all-cause mortality during follow-up in patients without diabetes mellitus. Odds ratios (OR) and adjusted odds ratios (aOR) are reported with 95% confidence intervals (CI).
